# IMPULSE Moment-by-Moment Test: An Implicit Measure of Affective Responses to Audiovisual Televised or Digital Advertisements

**DOI:** 10.3390/bs10040073

**Published:** 2020-04-05

**Authors:** Gemma Anne Calvert, Geraldine Trufil, Abhishek Pathak, Eamon Philip Fulcher

**Affiliations:** 1 Nanyang Business School, Nanyang Technological University, 50 Nanyang Avenue, Singapore 639798, Singapore; 2 Split Second Research Ltd., London E1 8FA, UK; geraldine.trufil@splitsecondresearch.co.uk (G.T.); eamon.fulcher@splitsecondresearch.co.uk (E.P.F.); 3 School of Business, University of Dundee, Dundee DD1 4HN, UK; a.z.pathak@dundee.ac.uk

**Keywords:** advertising, audiovisual content, implicit measure, advertising effectiveness, implicit reaction time, neuromarketing, YouTube, online experiments

## Abstract

IMPULSE is a novel method for detecting affective responses to dynamic audiovisual content. It is an implicit reaction time test that is carried out while an audiovisual clip (e.g., a television commercial) plays in the background and measures feelings that are congruent or incongruent with the content of the clip. The results of three experiments illustrate the following four advantages of IMPULSE over self-reported and biometric methods: (1) being less susceptible to typical confounds associated with explicit measures, (2) being easier to measure deep-seated and often nonconscious emotions, (3) being better able to detect a broad range of emotions and feelings, and (4) being more efficient to implement as an online method.

## 1. Introduction

An ideal test to measure the effectiveness of an advertisement is one that can provide a single number that captures its overall performance. This might then be used iteratively to optimize the creative content or to decide whether or not to proceed [1]. However, in practice, an advertisement’s effectiveness needs to be measured across several dimensions, as no single measure is sufficient. In addition, research suggests that an advertisement needs to elicit some form of emotional response that is compatible with the desired action [2]. The emotion elicited is important because it can influence perception, guide attention, influence memory retrieval, alter values and beliefs, communicate how we feel about others, and guide decision making [3], all of which can impact on how consumers react to the communication. Yet, detecting the precise emotion triggered by an advertisement is not a straightforward endeavor [4]. According to several reviews of the literature on the measurement of emotion, there is no single method that can successfully capture the broad range of emotional responses [5,6]. Each method captures only some aspect of emotion and often the method used itself is determined by the model of emotion one adopts.

Some theories posit that emotions exist as discrete entities. For example, categorical emotion theory proposes that an innate set of emotions exist universally across cultures (e.g., fear, anger, disgust, sadness, and happiness) and during maturation, we develop the more complex emotions of shame, embarrassment, contempt, and guilt [7]. The theory assumes that emotions are independent and so can be captured or represented on unipolar scales (i.e., any single emotion can either be present to a measurable extent or be absent). Alternatives to categorical theories are dimensional models of emotion in which specific emotions arise from at least two basic bipolar dimensions of affect: valence (pleasure vs. displeasure) and arousal (activation vs. deactivation). An example of this is the circumplex model of affect [8], which can describe ‘affect space’ across these two dimensions, with each emotional label located at some point in this affect space.

Equally important is the fact that emotion has a subjective, personal quality and at the same time, is partially observable by others and hence is objective. Subjective methods to measure emotion involve asking participants to explicitly state how they feel (after watching or listening to an advertisement) by rating it on a range of emotions that form a unipolar or bipolar scale. Examples of rating scales include the Positive and Negative Affect Schedule (PANAS [9]) in which participants are asked to describe the presence of 10 positive and 10 negative feelings or emotion labels on a scale of 1 to 5. This measurement can be used to indicate feelings in specific time frames such as now, yesterday, over the past month, and in general. A similar measure is the Differential Emotion Scale (DES [10]), which is used to assess 30 feeling words across 10 basic emotions. Examples of visual rating scales include the Visual Analogue Scale (VAS [11]), which involves marking a point on a line representing the emotion on a 100-point scale, and the Self-Assessment Manikin (SAM [12]), which is a nonverbal pictorial measure (that uses a cartoon’s facial and bodily expressions) along the dimensions of valence, arousal, and dominance. Scores on these three measures can be used to identify the emotion by using the circumplex model.

Self-reported affect can be useful for measuring global feelings over an extended period of time, for example, those that arise throughout the period of an advertising campaign. However, since such measures are often collected *after* the event, they are dependent on the accurate recall (which is often flawed) of one’s emotional states. Another problem is that such methods are not able to correctly identify the specific moments of an event (e.g., particular frames in a television commercial) when the emotion was elicited. It also assumes that the emotion elicited is fairly constant rather than continually fluctuating. Moment-to-moment fluctuations in affective experience have therefore been of interest and the dial test via a user interface was developed to overcome this potential shortcoming [13,14,15]. In the dial test, participants turn a dial clockwise or anticlockwise depending upon how they feel whilst watching or listening to a commercial. One problem with this measure is that only one or two emotions can be recorded at any given time as it is too cognitively challenging to report on more. One way around this is to use a movie clip or sound track multiple times. For example, Krumhansl [16] has used a dial or slider to record several emotions, (e.g., sadness, fear, happiness, and tension) by repetitive listening or by using different participants for each emotion. While the dial test is used on one dimension at a time, the EmotionSpace Lab [17] features a two-dimensional emotion-space model for movie clips and music. The participant moves a mouse or similar input device along a two-dimensional grid, with axes labeled as Valence and Arousal (similar to the circumplex model). Similar methods include EMuJoy [18], which can be used over the internet, and Feeltrace [19], which uses color coding in addition to the two dimensions.

These measures are clearly subjective, and may be contaminated by a number of biases, such as participants’ expectations about the experiment, demand characteristics of the study, the need to give an impression to the researcher (the social desirability bias), and the real problem of not being able to introspect accurately or to locate one’s feelings on an absolute scale. However, as a measure of current emotional state, self-report is likely to be valid in certain cases, at least in terms of emotional valence and level of arousal, which can occasionally account for a considerable portion of the variance [20].

Arguably, more objective methods might involve capturing one or more physiological measures (e.g., heart activity, electrodermal responses, and breathing rate). However, while such measures can provide strong indications of a person’s level of arousal and in some cases even valence (e.g., the phasic responses of heart rate), there is no clear agreement on the precise pattern of the physiological responses for each emotion felt. Instead, they have to be used in combination with subjective measures (see Poels and Dewitte [4] for a review). On the other hand, if specific physiological measures can predict certain parameters (e.g., sales success), then they clearly have great value. Yet it appears that no single method is able to do this reliably [21,22], although Venkatraman et al. [23] claim that the activity in the ventral striatum [measured using functional magnetic resonance imaging (fMRI)] can predict the effects of advertising better than the previous methods mentioned above. Another recent area of interest is facial coding, where facial microexpressions are classified into between four and seven emotions (depending on the specialist software being employed) attached to a video camera (or webcam). However, in practical terms, emotional facial expressions evoked while viewing advertisements tend to be infrequent, and facial coding systems can often make higher type II errors [24], especially in males who are less facially emotive [25].

## 2. Automatic Evaluations

An alternative approach is to examine the evaluative components of emotion. It has been argued that everything we encounter provokes a spontaneous or automatic evaluation on a range of dimensions (e.g., liking, threat, attractiveness, desire). These evaluations can take place spontaneously and nonconsciously, that is, without deliberate intention and often without our conscious awareness of those emotions [26,27,28,29]. Every automatic evaluation sets in motion the seeds of an emotional process and whether the evaluation results in a full-blown emotional response (i.e., a physiological change with a subjective feeling) is dependent upon its strength, its duration, the context, our motivation, and many other similar factors. The important point here is that the external stimuli (e.g., objects, products, places, television and radio advertisements) are value-charged and can give rise to an automatic evaluation.

In several versions of this theory, automatic evaluations apply value tags to the things we encounter and can subsequently lead to a basic emotion [4], such as excitement or fear, influencing the decision to approach or avoid and attack or retreat. However, in our version (IMPULSE test), the automaticity of an evaluation can reflect not only its basic or biological importance but also its social importance to the individual, and this includes higher-level evaluative dimensions (e.g., trust, trendiness, advocacy, reliability, authenticity, and originality). Equally important is that automatic evaluations can also have subsequent specific and nonconscious effects on the other cognitive processes, such as attention, perception, memory, and decision making [30,31], and behavior [27,32]. Automatic evaluations have also been shown to influence social conformity, face perception, interpersonal perception, emotional regulation, moral judgments, relationship formation and maintenance, stereotyping, and prejudice [33].

For the current paper, tapping into automatic evaluations can provide insight into the effectiveness of an advertising campaign in terms of how positively it is perceived, how memorable it becomes, and the extent to which it can affect subsequent decision making or purchase behavior. This is a potentially important route through which we might identify the effects of advertising. Fortunately, recent developments in implicit reaction time testing offer a way forward for doing this, and while it might not provide a single solution to the problem, it can provide insights in a language that creative developers, brand managers, and marketing managers can use. There have been considerable successes using this implicit approach to provide an objective measure of attitudes in consumer research [34], and in many aspects of emotions in general (e.g., [35,36]).

## 3. Affective Priming

Affective priming is a paradigm developed to measure automatic evaluations by comparing responses to emotionally congruent (vs. incongruent) presentations of stimuli [37]. In its original version, the task requires respondents to detect a word as having a positive or negative meaning by pressing one key on a computer keyboard for positive words (e.g., fun, happy) and another key for negative words (e.g., dull, sad) as quickly and as accurately as possible and within an established timeframe. After this task has been learned, in the second phase of the task, word ‘primes’ appear before the presentation of the target words. The prime itself might have a positive or negative meaning (e.g., joy or despair). It is found that when the prime and the target have the same valence (e.g., joy + happy), the time taken to detect the target (detecting that happy is a positive word) is faster than when the prime and the target have opposite valence (e.g., despair + happy). The psychological interpretation of this effect is that the presentation of a prime automatically triggers associations in memory, so that when an associated item is subsequently presented, its detection is facilitated by the preceding prime. On the other hand, when a nonassociated item is presented, its detection is inhibited. In short, congruent prime and target combinations facilitate and decrease reaction times to the targets while incongruent prime and target combinations inhibit and increase reaction times.

Of course, congruency is subjective and the test measures automatic evaluations unique to the individual. In the context of consumer behavior, if respondents have a strong sense of trust for a particular brand (e.g., MTV), then they will make positive automatic evaluations of trust when they see the MTV logo. This may act in a congruent way in an affective priming test when they see trust-related words (e.g., reliable, dependable, trusted) and in an incongruent way in response to words related to mistrust (e.g., mistrust, doubt, misgiving) as in Calvert et al.’s [38] study (see [38,39,40,41] for a similar approach on semantic priming using audio and visual stimuli).

IMPULSE is an affective priming test with an audiovisual clip running in the background, but still in full view. In this case, the movie content acts as the prime and the targets are words belonging to one of two categories. The two categories reflect two poles of a single attribute (e.g., trusted vs. not trusted) on an emotional dimension. The audiovisual clip, which is not directly part of the task, will nevertheless influence the speed and accuracy of responding [37,38]. Words that are congruent with how the clip makes the viewer feel (e.g., happy) are easier to identify, while words that are incongruent with the feelings (e.g., sad) are harder to identify. For example, a movie clip delivering trust in a brand should facilitate the detection of trust-related words and inhibit the detection of words related to mistrust. This test shows a way in which we can measure a viewer’s feelings towards a television commercial or indeed any type of audiovisual content. To our knowledge, this is the first of such tests to have been developed.

In the experiments described here, the first is a test of the validity of IMPULSE; if it is able to measure emotional feelings, then it should be able to detect some of the basic emotions in audiovisual (AV) content *known* to elicit those emotions. We used clips with a known targeted emotional response and compared self-reported emotions of respondents with those obtained from the IMPULSE test. In the second experiment, we applied the test to two commercials that attempt to elicit different emotions, and in the third experiment, we compared IMPULSE responses to a television commercial for a well-known beauty product on customers and noncustomers of the product, as a further test of validity.

## 4. Experiment 1

### 4.1. Method

IMPULSE was configured to measure a specific bipolar emotion or feeling, such as joy vs. sadness. The test consists of two phases: a baseline or control phase and an experimental phase. During the control phase, the reaction time task was presented with an emotionally neutral movie clip running in the background (or with no clip). In the experimental phase, the to-be-assessed movie clip was presented. In both phases, the same set of emotion words was presented in a randomized order and each word had to be categorized as either one feeling or its opposite (depending upon the emotion or attribute being measured). Words were presented at the rate of about one every two seconds. Response latencies during the control phase acted as the baseline values. In the analysis, response latencies during the experimental phase were compared with the baseline values on a moment-to-moment basis and in two-second segments.

The prediction was that emotion words that were congruent with the content of the movie clip would tend to be detected and correctly classified more quickly than incongruent emotion words. For example, when watching a happy movie clip, joy-related words should be correctly categorized more quickly than the baseline response time and sadness-related words should be correctly categorized more slowly than the baseline response time.

The first experiment was designed to pilot the method on basic emotions. We created four IMPULSE tests for each of the four video clips (each 64 seconds long and converted to Adobe Flash (.swf) format, so that they could be quickly downloaded onto the user’s computer). Each one assessed a single bipolar emotion: one measuring joy, a second measuring fear, a third measuring disgust, and a fourth measuring surprise. The movie clips were chosen to elicit those emotions. If our hypothesis that this method measures emotional responses is true, then it should provide an accurate measure in response to a highly emotionally charged dynamic stimulus (e.g., emotional AV clips). 

### 4.2. Participants and Materials 

Participants (n = 190) were recruited through a research recruitment company (Research Now) who provides monetary incentives for participation. Selection criteria included normal or corrected-to-normal vision, native English speaker, over 18 years of age. All subjects gave their informed consent for inclusion before they participated in the study. The study entitled “Using the implicit association test to measure positive affect” was conducted in accordance with the Declaration of Helsinki, and the protocol was approved by the Ethics Committee of the University of the West of England and agreement to participate was made via a consent form.

The clip chosen to elicit joy was called ‘*The best surprise military homecomings: Part three’* and consisted of military personnel returning home to their loved ones, and in most cases, a child (https://www.youtube.com/watch?v=wZvnONUMNoo). The movie clip chosen to elicit disgust was a scene from the movie *Trainspotting* and consisted of a man using a very dirty toilet. The clip chosen to elicit fear was a scene from the movie *Silent Hill* (*Pyramid Head scene* https://www.youtube.com/watch?v=BnaAM4mHzEY). The clip chosen to elicit surprise was a commercial for the launch of the TV channel TNT in which several unrelated and unusual happenings were staged on the street after someone pushes a large red button labeled, *Push to add drama* (https://www.youtube.com/watch?v=UM7EMzVaNCk). 

### 4.3. Word Sets

For the tests, we devised long lists of emotion words and asked a panel of 12 nonpsychologists to tick the words which they associated with each emotion (and their opposites). We selected the top eight words (and their opposites) for each emotion.

*Joy*: Happy, Pleased, Cheerful, Peaceful, Joyful, Delighted, Faithful, Glad, Sad, Sorrowful, Grieving, Miserable, Upset, Painful, Gloomy, Troubled

*Disgust*: Filthy, Foul, Sickened, Diseased, Dirty, Nasty, Revolted, Disgusted, Nice, Pleased, Attracted, Delighted, Good, Adored, Liked, Clean

*Fear*: Horrified, Terrified, Threatened, Scared, Nervous, Fearful, Afraid, Frightened, Calm, Warm, Kind, Friendly, Affectionate, Sociable, Great, Relaxed

*Surprise*: Astonished, Funny, Entertained, Creative, Energetic, Interested, Surprised, Excited, Disappointed, Outraged, Bored, Useless, Irritated, Dulled, Confused, Annoyed 

### 4.4. Explicit Ratings

To obtain explicit subjective perceptions of each movie clip, a PANAS-type self-reported scale was used and the items presented were *Happy, Disgusted, Fearful, and Surprised*, where each item had to be rated on a five-point scale. 

### 4.5. Procedure

Participants were presented with four tests, each with two movie clips. For each of the four tests, the same procedure was followed. Participants were told that they were going to perform a task that measured how quickly they could classify words on the screen. Before the main trials, participants were given 24 practice trials with a set of practice words not used in the main trials. The task was to detect the type of word that had been presented, and to press the “E” or “I” key on the computer keyboard corresponding to the word categories used (counterbalanced across the sample), as quickly and accurately as possible. Each practice word was randomly presented twice. Warning messages were given if a response was incorrect, that is, if two keys were pushed at the same time, or if no key was pushed within two seconds. The next trial appeared after a short intertrial interval (ITI) of 1500 ms. Following practice trials, participants were told that the next task would be very similar but this time a movie clip would be shown in the background. Participants were instructed to focus on the classification task, but were told that the video may interfere with this task. The presentation order of the set of words was randomized and the following were recorded: 1) the time taken to correctly classify each word in both phases, 2) the actual word presented, and 3) the timing of each word presentation.

Each movie clip was assessed on each of the four emotions resulting in 16 different IMPULSE tests. Each participant completed four of these tests for each movie clip, and using each of the four sets of emotive words once. To achieve this, participants were divided into four groups, with each participating in four different tests. This ensured that each group of participants (a) were shown one congruent pair, (b) were tested on all four movie clips, and (c) were tested using all four word sets. It also ensured that (a) all word sets were fully paired with each movie clip and (b) movie clips and word sets appeared in a counterbalanced order.

After each test, participants were asked to complete the PANAS, which indicated the overall emotion they felt whilst watching the movie clip in the second phase. Having completed this procedure for the first movie clip, it was repeated for the second, third, and fourth movie clips. Each test took between three and four minutes to complete and the whole study took between 12 and 16 minutes for each participant. 

### 4.6. Results

In preparing the data, we followed the method outlined by Fazio and Olson [37], and for each test a Facilitation Index (FI) was then computed for every word presented. If the word was a member of the relevant category (e.g., the word *Happy* in the *Joy* word set condition), the FI was computed as the baseline reaction time minus the main test reaction time; if the word was in an opposite category (e.g., the word *Sad* in the *Joy* word set condition), the FI was computed as the main test reaction time minus baseline reaction time. In this way, an FI greater than zero implies a response that is congruent with the emotion category and an FI less than zero implies a response incongruent with (or opposite to) the emotion category. 

#### 4.6.1. Joy Movie Clip

**Figure 1:**
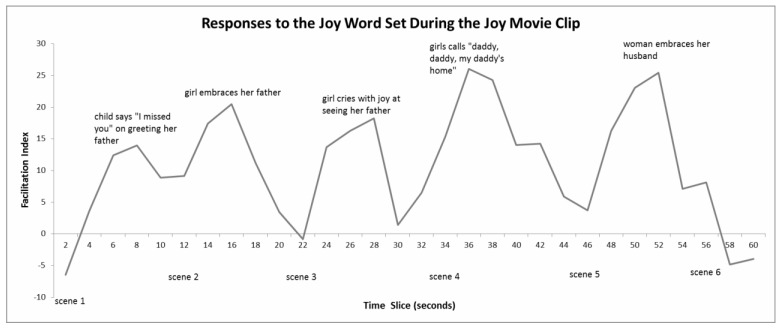
Three-point moving averages of facilitation indices for the Joy word set whilst viewing the Joy movie clip. Positive scores imply participants were experiencing joy, negative scores imply they were experiencing the opposite (sadness). The start points of each of the six scenes are indicated, as well as their peak indices and a brief description of the content at that point in time. Analysis indicated that the facilitation indices at the seconds 8, 14, 16, 24–28, 34−38, and 48−52 (all of the peaks) are significantly different from zero (in each case *p* < 0.05).

As predicted, there was an effect of the type of target word on the facilitation indices (*p* < 0.003). *Joy* words produced the highest FI (see Table 1). Moment-to-moment facilitation indices for *Joy* are plotted in Figure 1. Analysis of the explicit ratings of the Joy movie clip indicated that for participants, joy-related words were congruent with the content of the clip and sad words were incongruent. We reason that this implies that participants experienced joy whilst watching this clip. In fact, the explicit ratings corroborate this interpretation where participants confirm the way they felt (see Table 2).

#### 4.6.2. Disgust Movie Clip

**Figure 2:**
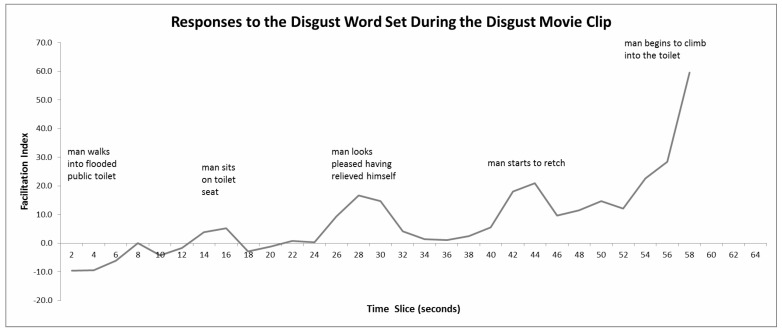
Three-point moving averages of facilitation indices for the Disgust word set whilst viewing the Disgust movie clip. Positive scores indicate disgust, negative scores indicate its opposite (attraction). The peak indices are also shown and a brief description of the content at that point in time. Analysis indicated that the facilitation indices at the seconds 28, 42−44, 54−58 are significantly different from zero (in each case *p* < 0.05).

Analysis showed an effect of the type of target word on the facilitation indices (*p* < 0.05), such that *Disgust* had the highest positive FI (see Table 1). Moment-to-moment facilitation indices for the *Disgust* movie clip are plotted in Figure 2. Analysis of the explicit ratings revealed that participants mostly felt disgust (see Table 2). These results show that at the implicit level, participants felt disgust, sadness (happy opposite), boredom (surprise opposite), but not fear or its opposite, and at the explicit level, they mostly felt disgust and a little surprise. 

#### 4.6.3. Fear Movie Clip

**Figure 3:**
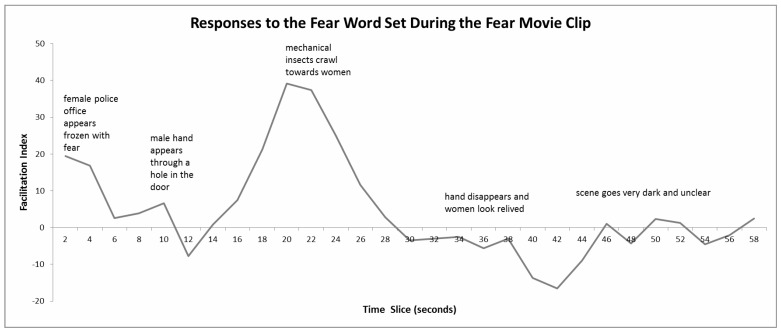
Three-point moving averages of facilitation indices for the Fear word set whilst viewing the Fear movie clip. Positive scores indicate fear, negative scores indicate fear opposite (serenity). A brief description of the content is shown at various points in time. Analysis indicated that the facilitation indices at the seconds 2, 4, 18−26, and 42 are significantly different from zero (in each case *p* < 0.05).

Analysis showed an effect of the type of target word on the facilitation indices (*p* < 0.05), such that *Fear* had the highest positive FI (see Table 1). Moment-to-moment facilitation indices for the Fear movie clip are plotted in Figure 3. These results indicate that at the implicit level, participants felt fear and sadness (happy opposite), while at the explicit level, little emotion was identified, although Frightened had the highest numeric mean. One interpretation is that respondents were less willing to admit to being frightened by a ‘scary’ movie clip (than they were to admit to feeling joy when watching a ‘happy’ movie clip), probably because showing fear is seen as a sign of weakness. Yet, despite this limitation, IMPULSE was still able to detect fear. 

#### 4.6.4. Surprise Movie Clip

Analysis failed to show any statistically significant effect of the type of target word on the facilitation indices (*p* > 0.05), with most facilitation indices close to zero (except *Joy* which had a negative value). Analysis of the explicit ratings shows that most participants felt moderately surprised, and little else was detected. 

### 4.7. Discussion 

These results show that overall, participants responded as expected in three of the four clips: they felt joy whilst viewing the Joy clip, disgust when viewing the Disgust clip, and fear when viewing the Fear clip, although not surprised when viewing the Surprise clip. Moreover, IMPULSE revealed the precise moments when these emotions rose and fell. The failure to detect surprise might be because 1) the word attributes poorly represented surprise, 2) the movie clip elicited little surprise, or 3) the test was not able to detect surprise as it is an extremely fleeting emotion. Further research would be required to determine this. Having tested the method against movie clips from various sources on specific emotions, the next experiment was designed to determine whether it could be used to detect general positive and negative affect in two different types of television commercials.

## 5. Experiment 2

### 5.1. Introduction

In this second experiment, we applied the method to two contrasting commercials. These were the Nikon *I Am Part of the World* as a positive and inspiring clip, and the *Cruelty to Children Must Stop* from the National Society for the Prevention of Cruelty to Children (NSPCC) as a negative and saddening clip (both clips of 60 seconds each). The target words were the same as those used in the Joy/Sadness word set in Experiment 1, and the task was to categorize each word as either positive or negative; all other aspects of the procedure were the same as in Experiment 1.

Participants (between the ages of 18 and 65 years) viewed either the positive or the negative commercial (n = 70 in each commercial) and were recruited through the same recruitment agency as in Experiment 1. As described previously, we measured the participants’ baseline reactions to the target words (Positive and Negative words) and then tested these attributes whilst they viewed the commercials. We then calculated the facilitation indices for every two-second slice of the clip, for each participant and for each commercial.

### 5.2. Results

**Figure 4:**
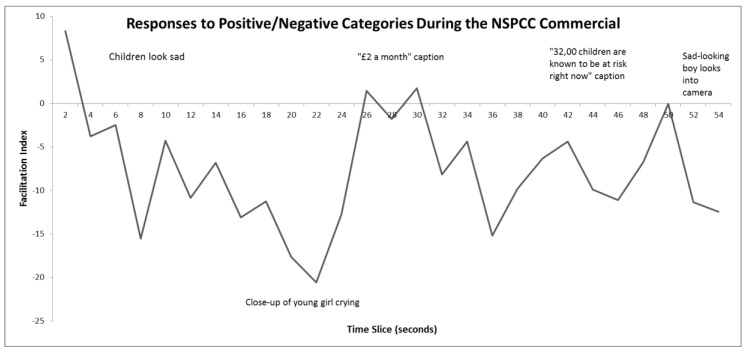
Three-point moving averages of facilitation indices for the Joy word set whilst viewing the NSPCC commercial. Positive scores indicate positive affect, negative scores indicate negative affect. A brief description of the content is shown at various points in time. Analysis indicated that the facilitation indices at the points 8, 16−24, 36−38, and 52−54 are significantly different from zero (in each case *p* < 0.05).

**Figure 5:**
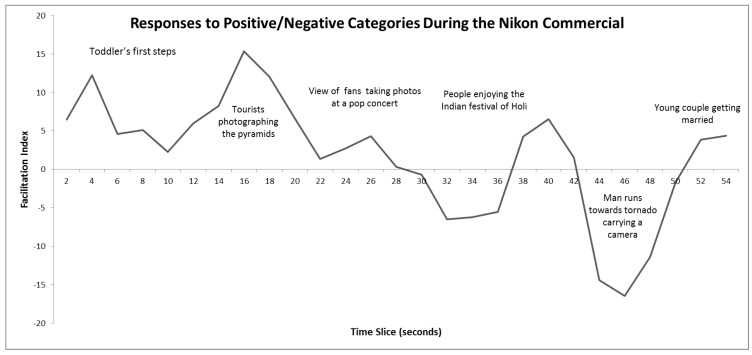
Three-point moving averages of facilitation indices for the Joy word set whilst viewing the Nikon commercial. Positive scores indicate positive affect, negative scores indicate negative affect. A brief description of the content is shown at various points in time. Analysis indicated that the facilitation indices at the points 16 and 44−48 are significantly different from zero (in each case *p* < 0.05).

Overall, the negative commercial elicited a response consistent with the negative affect (FI = −7.65; Figure 4) and the positive commercial elicited a response consistent with the positive affect, though much smaller in size (FI = 2.01; Figure 5). Explicit ratings confirmed the way participants felt (see Table 3). The moment-to-moment trace for the positive commercial shows that whilst it elicited positive feelings initially, these were gradually replaced by negative feelings as the commercial progressed (Figure 5). This commercial ends with a man with a camera running towards a tornado, which unexpectedly seems to have elicited negative affect in the viewers (one interpretation is that they feared for his safety). The negative commercial shows children looking upset. The captions indicate that this is a charitable appeal on behalf of the NSPCC. The moment-to-moment trace clearly showed that the emotional response in viewers was almost entirely negative throughout (Figure 4). There was a small positive peak around the middle of the commercial, which coincided with the caption “£2 a month” displayed over the NSPCC logo and a telephone number.

### 5.3. Discussion

The second experiment showed that when applied to television commercials, IMPULSE was able to detect the feelings elicited. It revealed almost continuous negative affect whilst watching the charity appeal and revealed mostly positive affect whilst watching the positive commercial. In the next experiment, we wished to examine this method for analyzing a higher-level emotion or feeling towards a television commercial. Very often, a commercial is designed to target a specific, more complex or higher-order feeling (e.g., trust, fascination, amusement) in order to enhance consumer interest/engagement with the brand and ultimately to change the purchase behavior.

## 6. Experiment 3

### 6.1. Introduction

In this experiment, we chose to examine *uniqueness* versus *ordinariness* in a *L’Oréal* commercial that featured the actress Lea Michele. The method was the same as in Experiment 2, except that the clip lasted for 32 seconds. The target categories were *Unique/Positive* and *Ordinary/Negative*, and the target words to categorize were *Unique, Special, Distinctive, New, Ordinary, Plain, Mediocre,* and *Common*. Furthermore, to validate this method as a predictor of purchasing intentions, participants chosen in this experiment were either customers or noncustomers of *L’Oréal*. 

Participants were 166 women recruited via a sample recruitment agency based in the USA and were tested online, with 60 users and 106 nonusers of *L’Oréal* products. As in the previous experiments, we measured the participants’ baseline reactions to the target words (*Unique/Positive* and *Ordinary/Negative* words) and then tested these attributes whilst they viewed the *L’Oréal* commercial. 

### 6.2. Results

**Figure 6:**
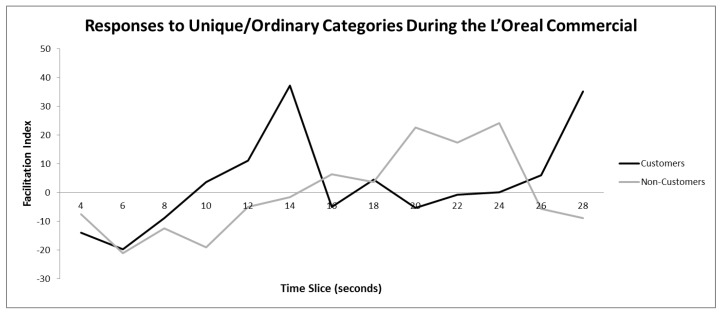
Trace of IMPULSE whilst viewing the *L’Oréal* commercial for customers of *L’Oréal* products and noncustomers. Positive scores indicate feelings congruent with Unique and/or Positive, negative scores indicate feelings congruent with Ordinary and/or Negative.

As with Experiment 1 and 2, we computed facilitation indices and in this case, an FI greater than zero implied a response that is congruent with *Unique/Positive* and an FI less than zero implied a response congruent with *Ordinary/Negative*. Overall, customers of *L’Oréal* products had higher FIs than noncustomers (*p* < 0.05). Indeed, this pattern of fluctuation differed between two groups and key differences occurred at time slices 14, 20–24, 28–30 (for each, *p* < 0.05; see Figure 6). 

Next, we broke down the facilitation indices into basic reaction times for each of the eight component target words and compared customers against noncustomers. This allowed us to determine precisely which target words were driving the positive customer response at t = 14 and t = 28. By examining this data, we see that at t = 14, customers positively associated *Unique* and *Distinctive* and negatively associated *Mediocre* and *Ordinary* with the content at this point in the commercial. Similarly, the late rise in positive customer response at t = 28 can be explained by positive associations of the content with *Unique* and *Special* and negative associations with *Common* and *Plain*. 

Similarly, breaking down the facilitation indices for noncustomers enabled us to account for the positive response between t = 20 and t = 24. Doing this revealed a positive association of the content between *Special, Distinctive,* and *New*, and a negative association of the content between *Mediocre* and *Common*.

Analysis of the explicit questions about the commercial showed that a significantly higher number of *L’Oréal* customers (when compared to noncustomers) agreed with the statements: 

*“The product seemed credible”* (68% vs. 47%),

*“I found the content engaging”* (57% vs. 44%),

*“I am inspired to make a purchase”* (22% vs. 6%),

*“I will tell others about it”* (15% vs. 5%),

*“It makes me like this product”* (28% vs. 18%).

Overall, the *L’Oréal* commercial had differential effects on *L’Oréal* customers and noncustomers. In general, customers reacted negatively to the male voice-over, and positively to actress *Lea* talking. Customers also reacted very positively to *Lea* using the phrase, *“Because you are totally worth it”* whilst noncustomers reacted very negatively to it, and these observations are consistent with what one might expect from these two groups. All viewers associated the moment when the male voice-over is listing potential hair problems at t = 6 with *Ordinary/Negative* (using the negative terms such as *“weak, limp, lifeless, dull,* and *straw-like hair”)*.

## 7. General Discussion

In three experiments, we show that the IMPULSE test is useful for determining the emotional responses to movie clips on a scene-by-scene basis, and therefore useful for evaluating the creativity and effectiveness of television commercials. We speculate that the test would also be useful for evaluating radio commercials, other animated forms of advertising (such as web-based advertising), and even political broadcasts and speeches. The test has several advantages over self-reported measures. Firstly, it is an indirect measure and hence not susceptible to the effects of social desirability or insincere responding. Secondly, it does not require the viewer to introspect or self-monitor when providing their response (such self-knowledge of emotions may not always be easy to access), and thirdly, the test obtains moment-to-moment scores rather than postevent scores. The test also has several advantages over physiological measures. Firstly, the test can be carried out online and participants are not required to visit a laboratory. This could save considerable resources in testing and make it easy to obtain a larger sample. Secondly, unlike physiological tests where only general information about the emotional response is yielded (such as the level of arousal), IMPULSE is able to detect highly specific emotions and feelings.

The kind of information obtained by IMPULSE can be useful for creative marketing purposes. In our third experiment, we revealed that whilst customers generally tended to take pleasure in hearing specific brand claims and straplines, noncustomers did not. This information is useful for marketers. Finer analysis of the individual attributes revealed even more specific information about those feelings (e.g., through attributes *special, distinctive, new)*; these are high-level evaluations detected at the specific moment at which they occur in the advertisement.

Face validity of the test is demonstrated by its ability to detect expected emotions (Experiments 1 and 2), and concurrent validity by its ability to distinguish between customers and noncustomers of a brand (Experiment 3). Further research can examine the extent to which the feelings measured by IMPULSE are linked to purchase intentions. In a future study, it would be useful to measure the predictive validity of this test and compare those with previously published research [21,22,42]. Finally, we believe this new method opens a new avenue of research for examining the effectiveness of AV advertising, not only in terms of the emotions elicited but also in terms of the specific feelings detected and how they might encourage the viewer to buy the brand, product, or service being advertised (e.g., Calvert et al. [43]).

## 8. Conclusions and Practical Implications 

The IMPULSE test outlined in this article was developed as a tool for measuring implicit affective responses that occur while viewing dynamic visual stimuli. The findings of this research show that the test can successfully capture a range of general emotions and specific feelings experienced when viewing a movie clip (or television commercial). The test does not require self-reported measures and can tap into feelings which are difficult to elicit (e.g., feelings about sensitive issues) or are deep-seated and difficult to measure. Another advantage of the test is that it is fully automated and can be conducted online. Further research is required to compare its predictive validity with other reported tests in the field.

## Figures and Tables

**Table 1:** Implicit responses: facilitation indices per target pair for each clip.

Targets	Joyous Clip	Disgusting Clip	Scary Clip	Surprising Clip
Joy	+11.7	−8.6	−5.4	−9.7
Disgust	−10.3	+6.1	+0.5	+1.3
Fear	−5.2	+1.3	+5.6	+0.5
Surprise	−0.7	−6.8	−1.9	−2.5

**Table 2:** Explicit responses: emotion ratings for each clip.

Emotion	Joyous Clip	Disgusting Clip	Scary Clip	Surprising Clip
Happy	3.7	1.5	1.6	2.5
Disgusted	1.1	3.9	2.3	1.5
Frightened	1.2	2.0	2.4	1.8
Surprised	2.2	2.8	2.3	3.1

**Table 3:** Explicit responses: emotion ratings for both commercials.

Emotion	Nikon Commercial	NSPCC Commercial
Happy	3.0	1.2
Sad	1.1	3.0
